# Concept and Development of an Electronic Framework Intended for Electrode and Surrounding Environment Characterization In Vivo

**DOI:** 10.3390/s17010059

**Published:** 2016-12-30

**Authors:** Stefan B. Rieger, Jennifer Pfau, Thomas Stieglitz, Maria Asplund, Juan S. Ordonez

**Affiliations:** 1 Laboratory for Biomedical Microtechnology, Department of Microsystems Engineering (IMTEK), University of Freiburg, Georges-Köhler-Allee 102, 79110 Freiburg, Germany; stefan.rieger.imtek@gmail.com (S.B.R.); thomas.stieglitz@imtek.uni-freiburg.de (T.S.); maria.asplund@imtek.uni-freiburg.de (M.A.); 2 Cortec GmbH, Georges-Köhler Allee 010, 79110 Freiburg, Germany; 3 BrainLinks-BrainTools Center, University of Freiburg, Georges-Köhler-Allee 79, 79110 Freiburg, Germany; 4 Freiburg Institute for Advanced Studies (FRIAS), University of Freiburg, Albertstraße 19, 79104 Freiburg, Germany; 5 Medtronic, Medtronic Eindhoven Design Center, Neuromodulation High Tech Campus 41, 5656 AE Eindhoven, The Netherlands

**Keywords:** thin-film microelectrodes, failure mechanisms, pH measurements, impedance measurements, temperature measurements, stress measurements, neural implant

## Abstract

There has been substantial progress over the last decade towards miniaturizing implantable microelectrodes for use in Active Implantable Medical Devices (AIMD). Compared to the rapid development and complexity of electrode miniaturization, methods to monitor and assess functional integrity and electrical functionality of these electrodes, particularly during long term stimulation, have not progressed to the same extent. Evaluation methods that form the gold standard, such as stimulus pulse testing, cyclic voltammetry and electrochemical impedance spectroscopy, are either still bound to laboratory infrastructure (impractical for long term in vivo experiments) or deliver no comprehensive insight into the material’s behaviour. As there is a lack of cost effective and practical predictive measures to understand long term electrode behaviour in vivo, material investigations need to be performed after explantation of the electrodes. We propose the analysis of the electrode and its environment in situ, to better understand and correlate the effects leading to electrode failure. The derived knowledge shall eventually lead to improved electrode designs, increased electrode functionality and safety in clinical applications. In this paper, the concept, design and prototyping of a sensor framework used to analyse the electrode’s behaviour and to monitor diverse electrode failure mechanisms, even during stimulation pulses, is presented. We focused on the electronic circuitry and data acquisition techniques required for a conceptual multi-sensor system. Functionality of single modules and a prototype framework have been demonstrated, but further work is needed to convert the prototype system into an implantable device. In vitro studies will be conducted first to verify sensor performance and reliability.

## 1. Introduction

The development of implants with a higher density of stimulation (or recording electrodes) has become a key endeavour in neural engineering with the aim of accurately targeting specific neural structures and thereby increasing efficiency and reducing side effects. More densely spaced electrodes increase spatial resolution, allowing more accurate recording and more selective stimulation therapies to be applied. Miniaturization of these electrodes developed along with the transfer from precision mechanics to micromachining technologies, lead to the development of high-density thin-film electrode arrays. Such electrode arrays have been successfully implemented in retinal prostheses, electrocorticography (ECOG) arrays and cochlear implants [1].

Thin-films used for implantable applications are known for their failure mechanisms related to delamination, cracking, corrosion and dissolution. These failure mechanisms have a greater prevalence when the electrodes are used for electrical stimulation of tissue. Different from bulk electrodes, thin film electrodes reach their end of use lifetime faster. There is just insufficient material to withstand up to decades of deteriorating processes. These processes arise from the environmental conditions and it is of the utmost importance to be able to understand these at their roots to allow neural interface architects to develop a system that can survive the use conditions. Furthermore, the environmental conditions at different parts of the body can vary, particularly as a response to the implantation of a foreign body. While thin-film devices have shown an advantage in terms of higher selectivity, more controlled therapeutic delivery and less invasiveness, their lifetime remains limited and below the expectations required for medical device approval.

Standard electrode materials include platinum (Pt), gold (Au), iridium (Ir), palladium (Pd) and rhodium (Rh) as well as their alloys such as platinum-iridium (PtIr) and iridium oxide (IrOx). In addition to being biocompatible, materials used for electrical stimulation must be corrosion resistant, allow for reversible charge transfer [1] and have a high charge injection limit [2,3,4].

Characterization of: (a) the mechanical effects on the system; (b) the environmental condition (tissue response); and (c) the electrode-electrolyte processes during electrical stimulation and correlating all these to better understand the failure mechanisms limiting the lifetimes of these thin-film electrode arrays [2] is a vital part of translational research to establish their application in clinical practice [3].

As chronic in vivo electrode studies have reported variable results and differences to in vitro data in the past [3,5], there has been a growing need to develop systems to evaluate implanted microelectrodes with the aim of determining parameters such as charge injection limits, measuring temporal changes of material parameters and to develop charge injection protocols that limit polarization to stay within safe electrochemical limits in vivo [3]. Given that for a constant charge density the voltage produced across a metal electrode is typically inversely proportional to its surface area [6], such systems become vital for safely developing smaller electrodes.

State-of-the-art electrode characterization is performed in a laboratory environment, requiring an enormous effort to achieve a scientifically significant in vitro evaluation. The alternative of this method would be to perform in vivo studies, where parametric testing is performed by end-point analysis of the electrodes and surrounding tissue. Because of the large number of animals required for those in vivo trials, such an approach would be ethically questionable and extremely onerous. To provide more realistic and reliable data, the trend in biomedical sensor applications is to develop real-time and long term stable readout systems [7,8,9,10] for online monitoring of implanted systems.

Unlike current biomedical sensor implementations to measure temperature [7,9,11], pH [11,12], impedance [10,13] and redox reactions by cyclic voltammetry [7], this paper describes the design, development and prototype testing of a multi-sensor system to investigate failure mechanisms observed in thin-film electrode arrays (such as dissolution of materials, delamination of coatings, and fracture of functional metal layers) by investigating the interaction between stimulation induced electrochemistry and the electrode material in vivo. In comparison to standard sensor systems described in literature to monitor physiological signals [7,10,11] the proposed system is specifically designed to characterize electrodes and perform recordings during stimulation pulses. A particular goal of the research is to better understand the effect of neural intervention on the tissue and how the related phenomena affect the material-tissue interface of the electrodes in electrolytes on a chemical, biological and mechanical level. The ultimate aim is to extend the lifespan of active implantable devices with thin-film based microelectrode arrays.

During electrical stimulation, irreversible faradic processes can result in a drastic shift in pH in the vicinity of the electrode surface [3]. This shift in pH cannot only put the electrode materials at risk, but it can trigger undesired tissue damage or provoke a biological response. To stay within safe bioelectrical stimulation limits, it is important to carefully select the charge injection waveform and amplitude to prevent the onset of irreversible Faradic processes. It has also been shown that cells attached to the electrode array can further induce a local pH shift in the area between the cells and the electrode as a response to either cell attachment to the electrode or an applied electric potential on the electrode [14]. For this reason, pH monitoring should be done as close to the electrode surface as possible to be able to detect these processes.

Characterization techniques, such as impedance spectroscopy, provide insight into changes at the electrode-tissue interface during continuous electrical stimulation and can provide a basic understanding of the biological response to the electrode from the host environment. Furthermore, mechanisms resulting in electrode dissolution in vivo are not fully understood [6]. Even though models representing the electrochemical behaviour of platinum and platinum derivatives have been established in the literature for bulk materials, further understanding of the mechanical effects on the surface of the thin-film layers at different electrochemical potentials is still required [15,16]. In thin-film metallization, surface effects, such as oxidation, play a significant role [15]. Measuring the resulting stress induced into the material during electrical stimulation and from environmental exposure in vivo, provides one method of understanding these effects. An implantable sensor to measure temperature variations around an electrode could provide insight into the degree and duration of inflammation reactions that come along with a temperature increase from the host environment and could potentially also be used to monitor heating caused by electrical or eventually optogenetic stimulation [17]. Cyclic Voltammetry (CV) and transient potential measurements allow electrochemical characterization of diverse electrode processes including their reversibility, material changes, charge injection as well as their electrode-electrolyte interface. As these characterization methods suffer from external influences, such as temperature, electrolyte conditions and gassing, data reproducibility is difficult. So far, combinations of electrochemical and physical parameters (temperature, mechanical stress) cannot be recorded continuously within a single implantable device.

The work presented here illustrates the concept and development of a compact framework for a multi-sensory array with the ability to measure pH, impedance, stress, temperature, cyclic voltammetry and transient behaviour of thin-film platinum and platinum group metal electrodes near the electrode surface and under functional stimulation conditions. This reduces the number of single experimental setups by combining multiple measurements (allowing multiple parameters to be measured simultaneously), thus reducing measurement variations between different setups and allowing experiments to be done in vivo and not in separate test mediums. The ultimate goal is to measure and correlate the effects to achieve a better understanding of the interactions between the same experiments, enabling a causality analysis. The framework is designed as an analysis tool for implanted microelectrodes for research purposes. In addition, it is implemented to be low-cost, low-power, modular (each subsystem is implemented in a different module) to allow for customization, and have customizable data read-out for tailored experiments. The system will be further integrated with a fully customizable biphasic waveform stimulator. The stimulator design, however, is not covered in this paper.

## 2. Materials and Methods

**Figure 1:**
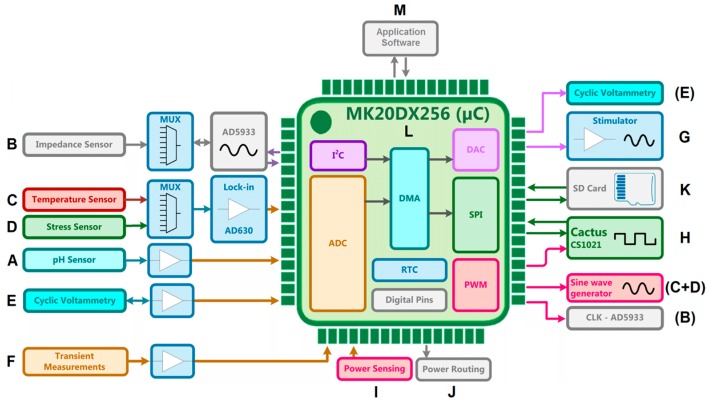
Block diagram of the MUlti-SEnsor array (MUSE) system composed of the following functional modules: A—pH sensor; B—impedance sensor; C—temperature sensor; D—stress sensor; E—cyclic voltammetry module; F—transient voltage analysis module; G—electrical stimulator; H—Cactus Semiconductor CSI021 (Cactus Semiconductor, Chandler, AZ, USA) stimulator (secondary stimulator); I—power monitoring to monitor the system; J—intelligent power routing to reduce power consumption; K—data storage (SD card); L—microcontroller and M—application software to control the system.

The measurement system was designed in a modular way including functional modules for a pH sensor, an impedance sensor, a temperature sensor, a stress sensor, a cyclic voltammetry module, a transient voltage analysis module, an electrical stimulator (allowing customizable waveforms), a module for power monitoring, a power routing module to activate/deactivate subsystems and data storage on a SD card (Figure 1). A Teensy 3.2 development board (PJRC.COM, Sherwood, OR, USA) embedded with a MK20DX256 32-bit ARM Cortex-M4 microcontroller (Freescale Semiconductor, Austin, TX, USA) was used to control the various modules and record the data. The system was designed to work with two multi-sensor arrays, one for the working electrode (WE) and one for the counter electrode (CE), allowing the effects on both electrodes to be measured simultaneously. The sensor related modules will be addressed individually in the following sections.

**Figure 2:**
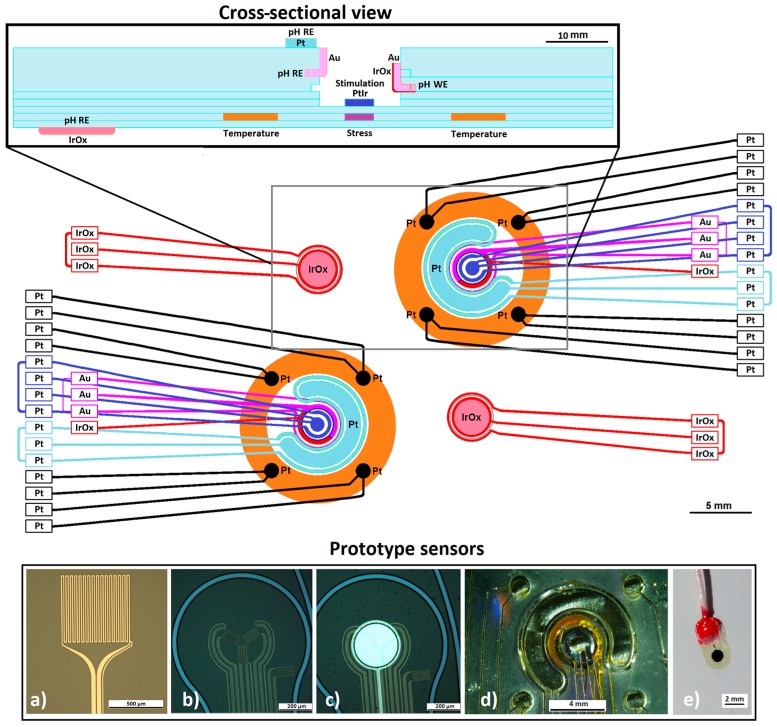
Conceptual sketch of the dual multi-sensor array configuration. A cross-sectional view of the array is shown (**top**). It depicts a PtIr stimulation electrode (**blue**) positioned above a stress strain gauge (**purple**) as well as surrounded by an IrOx sputtered on gold pH working electrode (WE) (**red**), an Au (**pink**) and Pt (**turquoise**) reference pH electrode (RE) and four Pt impedance electrodes (**black**). The temperature strain gauge (**orange**) is positioned around the electrodes and is prototyped with the strain gauge shown in (**a**) [17,19]. The PtIr stimulation electrode is positioned directly above the stress strain gauge depicted in prototype (**b**,**c**) [20]. Prototype (**d**) shows a picture of the prototype multi-sensor array with the four Pt impedance electrodes surrounding the pH and stimulation electrodes. The pH electrodes allow a differential measurement to be made between the working electrode (IrOx) and reference electrodes (Au electrode, semilunar shaped Pt electrode and more distant IrOx for pH measurement of the surrounding medium, intended to be insensitive to stimulation) while stimulating via the PtIr electrode is performed. The pH sensor used to verify the pH electronics is shown in (**e**).

The concept of the multi-sensor array consists of various sensor prototypes and a stimulation electrode, which are intended to be integrated into a final thin-film based sensor-array. The idea behind the sensor array was to arrange the various sensors around the stimulation electrode (blue electrode in Figure 2) to observe and characterize electrode behaviour and monitor the surrounding medium. These prototype sensors include a Pt thin-film temperature sensor (Figure 2a), a Pt thin-film stress sensor (Figure 2b,c), a laser-patterned foil and silicone rubber electrode array for pH and impedance measurements (Figure 2d) and an additional thin-film sputtered IrOx thin-film pH sensor. The electronic framework is not reliant on a specific sensor or electrode design, but can be used with any pH sensor, standard strain-gauge based stress or temperature sensor, impedance electrode and stimulation electrode. Before approaching a pure thin-film (wafer-level micro machined) electrode, the electronics for the impedance and pH measurement concepts were tested by using a simpler electrode manufacturing process, which involved laser-patterning of foil and silicone rubber [18] (Figure 2d). For this prototype foil-based array, we used PtIr foil for the stimulation electrodes, Pt foil for the impedance electrodes and IrOx sputtered on Au foil for the active pH electrode and Au as well as Pt foil for the pH reference electrodes (REs). With this approach, a cheaper and simpler test device could be used for validation compared to creating a more complex miniaturised micro fabricated device. Electrode materials were selected as typical candidates of active implants (cardiac pacemaker, cochlear and neural implants). The strain gauges implemented for detection of temperature and mechanical effects were not included in this silicone rubber version of the test electrode system.

### 2.1. pH Measurement

For accurate and stable pH sensing, high input impedance is crucial in reducing polarizing effects on the sensing electrode. For this reason, a high input impedance amplifier (>10^14^ Ω/pF) [21] is required to reduce bias currents through the electrodes. The INA116 differential instrumentation amplifier (Texas Instruments, Dallas, TX, USA) offers this high impedance with a high voltage headroom (±18 V) and ultra-low input bias currents of only a few femtoamperes [21].

**Figure 3:**
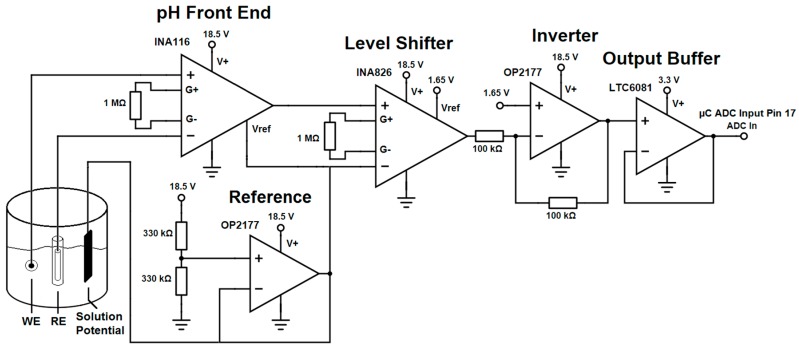
Schematic of the basic functional circuit of the developed pH readout system. WE: working electrode, RE: reference electrode.

This single supply IC allows a differential voltage to be measured without defining one of the pH electrodes to a fixed voltage such as ground. Additionally, the IC has integrated guard rails to reduce input leakage currents into the amplifier. For this application, the guard rails were connected from the amplifier inputs down to the physical sensor. The front end of the pH readout system (see Figure 3) was comprised of the ultra-low input bias current amplifier INA116 with a defined high voltage mid supply reference voltage provided by an OP2177 (Analog Devices, Norwood, MA, USA) reference operational amplifier. The signal chain was completed by level shifting the pH signal from a bias around the full voltage mid-supply (9.25 V) to a low voltage mid-supply (1.65 V), allowing the signal to be scaled down, inverted and buffered to a range from which it can be recorded with an Analog-To-Digital converter (ADC) integrated in the microcontroller. The final buffer ensured that the input was limited to 3.3 V to not exceed the ADC input voltage.

The purpose of the presented setup is to measure the pH level and changes of it during a stimulation pulse by placing the working/recording (IrOx) and reference pH electrode (Au or alternatively Pt) as close to the stimulation electrode as possible (Figure 2—cross-sectional view). The magnitude of the pH changes during single stimulation phases [3], or from an accumulated pH gradient over multiple stimulation cycles, is highly dependent on the proximity to the electrode surface. In contrast, the more the pH electrodes are exposed to the stimulation current flow, the more they will be exposed to high degrees of leakage currents, resulting in noise and potential drifting. The INA116 has a limited common mode input voltage range (V+ = −2) and (V− = +2.4) for the positive and negative rails, respectively. Therefore, these measurements require the solution to be biased with a mid-supply potential.

As the determination of the absolute pH value in an in vivo environment is difficult (due to noise, sensor drifting and the inability to recalibrate the pH sensor), we implemented a differential setup with a second IrOx pH electrode positioned on the backside, opposite to the stimulation electrode (Figure 2—cross-sectional view). Using a differential signal in this way allows the stimulation induced pH changes to more easily be discerned from those of the surrounding medium and should reduce stimulation artefacts. Notably, the relative changes in pH caused by the stimulation are of importance, hence these measurements are robust against sensor drift over time.

To validate the electronics, the electrode configuration was tested in a phosphate-buffered saline solution by measuring a sputtered iridium oxide pH recording electrode [22,23] (Figure 2e) versus a standard Ag/AgCl reference electrode in a two electrode setup. The open circuit potential of the pH setup was measured for an increasing and decreasing (reverse order) pH reference solution. The duration of the recording for each test cycle was two minutes, while one minute was allocated to move the electrode between reference solutions (pH 5.9 to 8.5). A digital multimeter (Agilent 34405A, Santa Clara, CA, USA), used with a real-time readout implemented in the software BenchVue (Keysight Technologies, Santa Rosa, CA, USA), was used as a reference measurement to compare to the results of the custom-built pH front-end. To evaluate the stability of the custom-made pH front-end over time, the same pH series was measured over a two-hour period. However, for this experiment the signal was recorded for one minute while one minute was allocated to move the electrode between solutions. To determine the hysteresis behaviour, the average pH from the descending and ascending pH solutions were displayed and the resulting trendlines were calculated.

### 2.2. Impedance Spectroscopy

**Figure 4:**
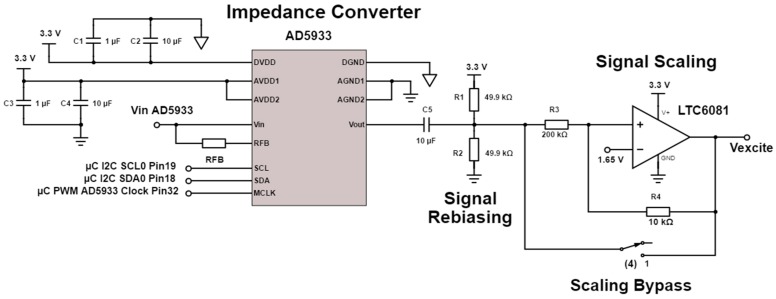
Circuit schematic of the AD5933 impedance analysis chip and signal scaling stage.

**Figure 5:**
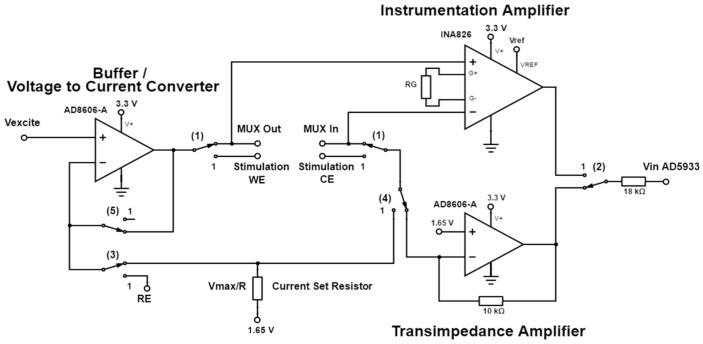
Impedance excitation circuit allowing voltage (two or three electrode setup) or current (two or four electrode setup) excitation of the system under test. (The current excitation signal in galvanostatic mode is defined using the current set resistance RSet = Vexcite_max/Iexcite_max).

The AD5933 (Analog Devices) low power, high precision impedance converter IC is capable of measuring both magnitude and phase of a complex impedance across a large frequency spectrum. With a rated system accuracy of 0.5% and a frequency resolution of less than 0.1 Hz, the AD5933 can perform an accurate impedance measurement over a small frequency range (±a few percent of the calibration frequency) [24]. The accuracy decreases over a large frequency range, requiring multiple recalibration stages. Using the IC’s standard configuration [24], the IC can analyse a frequency spectrum from 1 kHz to 100 kHz for an impedance range spanning 1 kΩ to 10 MΩ using the integrated internal clock. With more advanced circuitry [24], (Figure 4 and Figure 5) the impedance range can be extended down to 100 Ω. Furthermore, the frequency range can be stably extended down to as low as 20 Hz by adjusting the external clock frequency. The corresponding frequency for each frequency range (Table 1) needs to be separately defined during a multi-range frequency sweep.

The output voltage of the IC can be selected from a defined range (Table 2). Each excitation amplitude has a different DC bias voltage. The excitation signal therefore has to be re-biased back to the low voltage mid-supply level of 1.65 V by using a high-pass filter (*C5*, *R1* and *R2*) as shown in the circuit schematic (Figure 4) [25].

An important consideration is to keep the excitation voltage amplitude in the range of 10–50 mV to avoid polarizing the electrodes during the impedance measurements. For this reason, a signal scaling amplifier is required to reduce the signal amplitude by a factor of 0.05 (as set by resistors *R3* and *R4*). The gain of the internal transimpedance amplifier of the AD5933 was set with *R_FB_* (set to 18 kΩ) to provide unity gain.

**Figure 6:**
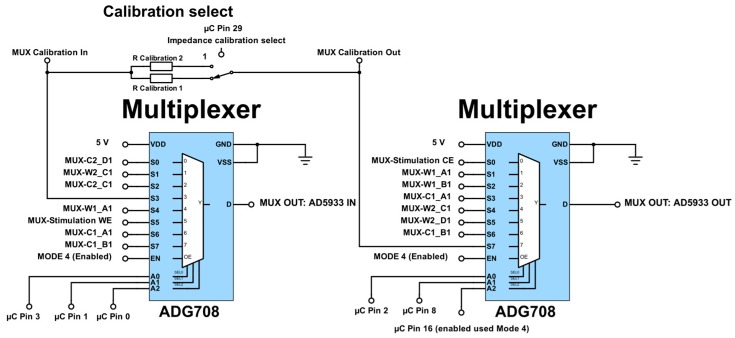
Circuit schematic showing the source multiplexing and the calibration resistance selection. Analog switch inputs A0 to A2 allow the different electrode configurations for the impedance measurement. Designation 1 and 2 correspond to the working and counter electrode, respectively.

The proposed circuit was designed to provide basic potentiostatic measurements in a two or three electrode setup. Alternatively, the setup also allows galvanostatic measurements to be made with a two or three electrode setup (implemented in Figure 5). The state of the excitation mode can be selected using switches (1)–(5), as shown in Table 3. Switch (1) sets the excitation signal across the stimulation working and counter electrode or across the multiplexed electrodes (Figure 6).

**Figure 7:**
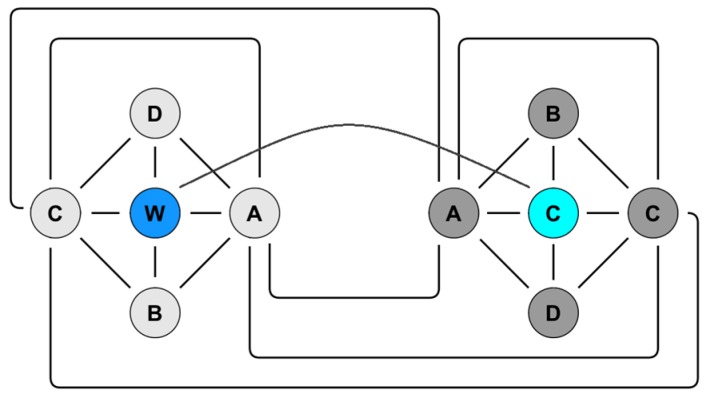
Possible impedance measurements for two multi-sensor arrays. Shown on the left is the working multi-sensor array with four impedance electrodes (A–D) located around the stimulation electrode (W). On the right is the counter multi-sensor array likewise with four impedance electrodes (A–D) located around the stimulation counter electrode (C). Each connecting line represents a possible impedance measurement option.

Different preselected configurations of impedance measurements can be performed (Figure 7). Each line represents a possible electrode pairing for impedance measurement. The setup potentially allows an impedance mapping around, across and between the stimulating electrodes, which for an implanted sensor array should provide insight into the resistivity and hence the degree of tissue encapsulation.

In the four electrode setup, the developed potential across the multiplexed electrodes is recorded by an INA826 instrumentation amplifier. Therefore, by selecting the appropriate impedance electrodes, a classic four electrode setup can be realized with both impedance electrodes “A” (Figure 7). To improve gain performance, an external transimpedance amplifier (AD8606) was used to replace the internal transimpedance amplifier of the AD5933. As the output of the AD5933 had an output impedance that varied with the selected output voltage range, the buffer stage (AD8606) provided a lower and mode independent output impedance path allowing measurement of resistances as low as 100 Ω. To be able to measure small current amplitudes such as those found in electrochemical measurements, the gain of the instrumentation amplifier was set to 1000 (*R_G_* = 49.9 Ω). After each frequency point measurement in a frequency sweep, the real (*Re*) and imaginary (*Im*) output registers of the AD5933 had to be read to calculate the impedance magnitude and phase of the system under test. The magnitude code (*M*) and phase (∅) are determined by using the equations defined in the AD5933 datasheet [24]. Even though the AD5933 datasheet suggests a single point calibration method [24], for truly accurate measurements, the AD5933 should be calibrated using a two-point calibration to account for voltage offsets inherent in the system. The phase of the system should be recalculated at every frequency point [24]. For system calibration, the magnitude and phase were measured across the two switchable resistors (Figure 6). These resistances should be chosen to represent the maxima and minima of the resistance range under investigation. With this calibration method, the system phase was determined using the real (*Re*) and imaginary (*Im*) output registers as:

(1) $$\Delta_{system}=0.5\times \left(Arctan\left(\frac{Im_{R_{1}}}{Re_{R_{1}}}\right)+Arctan\left(\frac{Im_{R_{2}}}{Re_{R_{2}}}\right)\right)$$

The actual impedance *Z* was determined from the magnitude *M* for each frequency point in the sweep:

(2) $$Impedance\;Z=\frac{\frac{1}{M}-\left(\frac{1}{M_{2}}-\frac{\frac{1}{M_{2}}-\frac{1}{M_{1}}}{R_{2}-R_{1}}\times R_{2}\right)}{\left(\frac{\frac{1}{M_{2}}-\frac{1}{M_{1}}}{R_{2}-R_{1}}\right)}\;\to \;x=\frac{y-c}{m}$$

*M*_1_ and *M*_2_ are the magnitudes measured with the calibration resistances *R*_1_ and *R*_2_ over the entire range of the frequency sweep, respectively. Furthermore, to remove the phase superimposed by the measurement circuit, the phase of the system under test was determined for each frequency point as follows:

(3) $$\emptyset_{SUT}=\left(\emptyset_{unknown}\;-\;\Delta_{system}\right).$$

To initially test the performance of the impedance measurement system, a characteristic curve outlining the magnitude code and phase versus applied frequency was performed for a range of resistors spanning 100 Ω to 39 kΩ. The phase was calibrated over a single resistor or alternatively was averaged over a pair of resistors (more accurate) to remove the system phase from the calculated phase as shown in Equations (1) and (2). To fully assess the calibration steps required to calibrate the impedance measurement system, the inverse magnitude versus resistance characteristic was investigated over a large selection of E12 resistors. For evaluation of the AD5933 setup, the values of a series of E12 resistors (eleven resistors ranging from 100 Ω to 39 kΩ) were measured using the proposed system and compared to a four-point LCR meter (ELC-3131D, Escort Instruments Corp., Hsin Tien, Taiwan) to determine the accuracy of the system.

**Figure 8:**
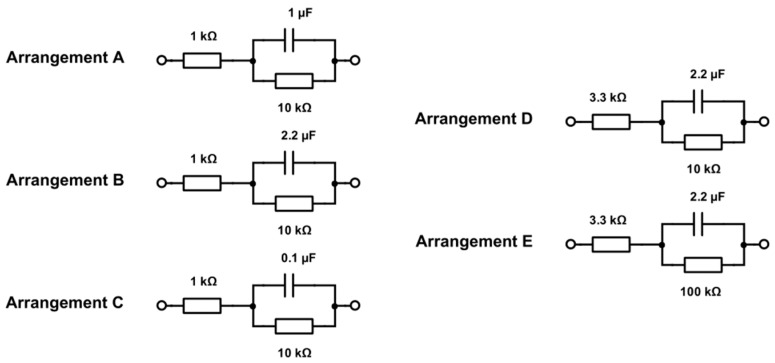
Five physical equivalent models (A—E) for characterization of the measurement setup and its calibration.

As a validation of the AD5933 impedance system, the impedance spectroscopy of an arrangement of five equivalent electrode models composed of a resistor in series to a resistor in parallel to a capacitor (Figure 8) was recorded using the two-point measurement and compared to a commercially available potentiostat/galvanostat (Metrohm Autolab PGSTAT302N, Utrecht, The Netherlands) with an embedded impedance spectroscopy module. As a final validation of the system, a 0.85 mm diameter platinum electrode, produced identically to the prototype impedance electrodes (Figure 2d), was characterized in a three-electrode setup.

### 2.3. Stress/Temperature

As both temperature and stress measurements can be performed using a Wheatstone bridge arrangement, the measurement electronics was integrated into a single readout system by multiplexing the inputs of the various sensors. To achieve reasonably fast measurements for each sensor connected to the system, separate Wheatstone bridges and calibration electronics were used for each sensor. This eliminates the need to recalibrate the bridge separately for each sensor as would be the case in a single Wheatstone application with multiplexed inputs, but this requires one calibration circuit for each channel. The developed system was designed to be interfaced with two multi-sensor arrays (one for the working electrode and one for the counter electrode) requiring eight Wheatstone bridges. Six bridges were required for two stress sensor rosettes (with each rosette containing three stress sensors at 60° angular separation), while an additional two were required for the single temperature sensor in each array. For the amplification stage of the readout system a lock-in amplifier was selected as a viable solution for the following reasons:

1. The changes in resistance are very small (<1 Ω) and the signal is partially or completely hidden in noise.
2. The lock-in feature of the amplifier allows stimulation induced interference from the proximity to the stimulation electrodes to be filtered out.
3. Being able to measure a signal with a high signal to noise ratio, allows the lock-in amplifier design to measure physical sensors in a noisy in vivo environment, where movement artefacts and electrical interference (such as those from cardiac activity) are expected.
4. Potentially lengthy lead wires passing through a fluid medium could also lead to considerable interference and noise, which can be filtered out using the lock-in amplifier design.

A sinusoidal signal (having less harmonics than a square wave) was chosen to excite the Wheatstone bridges and to drive the control input of the lock-in amplifier. A high recording resolution can be achieved even within a typical stimulation pulse by using a considerably higher excitation frequency than the stimulation frequency. A sinusoidal excitation frequency of 10 kHz provides a suitable choice.

**Figure 9:**
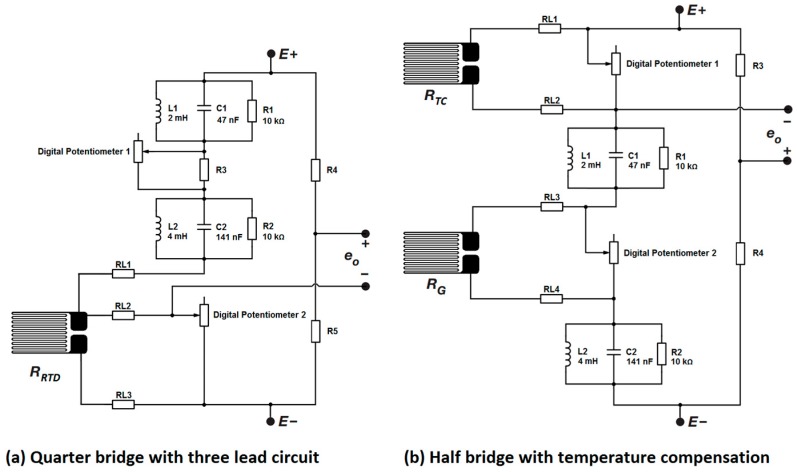
Circuit schematic of the Wheatstone bridge arrangements for (**a**) the temperature sensor and (**b**) the stress sensor. Digital potentiometers 1 and 2 are used to balance the resistive component of the bridge, while a dual parallel RCL arrangement is used to balance the imaginary component of the bridge.

Two modified Wheatstone bridge arrangements were designed for the temperature and stress sensors (Figure 9). The modification to the standard Wheatstone bridge arrangement allows balancing of the sensor bridge to the reference bridge, thus ensuring the Wheatstone bridge remains within its linear range. Since the sensors will be exposed to a fluid medium and will have potentially lengthy lead wires, both the temperature and stress sensors are expected to develop stray capacitances, which even at picofarad ranges, are enough to imbalance the sensor bridge and saturate the amplified output signal. The circuit allows the resistive component of the bridge to be rebalanced using digital potentiometers 1 and 2, while the two RCL arrangements provide a means of matching the reactive components of the bridge by allowing the system to be calibrated using the excitation frequency.

The reactive correction (matching of the imaginary part of the impedance) was simulated with NI Multisim (National Instruments Electronic Workbench Group, Austin, TX, USA). The fixed capacitance and inductance values determine the resonance frequencies of the two peaks, while the fixed resistance value effectively determines the peaks width. Although this method is robust against component tolerances, it is dependent on component drift. Hence only COG/NPO capacitors should be used as they are more stable than conventional ceramic capacitors.

**Figure 10:**
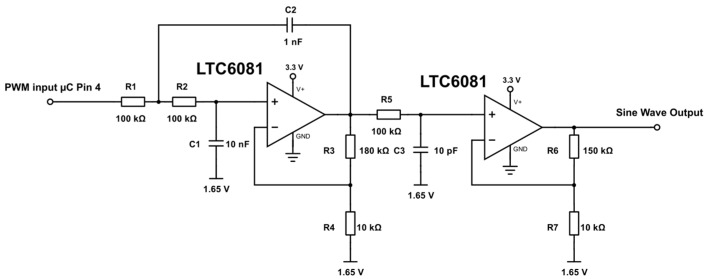
Circuit schematic of the square wave to sine wave converter based on a second-order Sallen-Key low-pass-filter (left amplifier) and active low-pass filter (right amplifier) design.

The sinusoidal excitation signal for the Wheatstone bridges was generated by passing a Pulse Width Modulation (PWM) signal from the microcontroller through a second-order Sallen-Key low-pass filter (Figure 10) [26]. The microcontroller PWM allows for single Hertz resolution and phase correction between the two bridges to within a hundredth of a degree. Phase correction to be controlled by changing the PWM frequency provides a faster calibration compared to just using a standard all-pass filter, which would require a digital potentiometer to be programmed for each correction.

**Figure 11:**
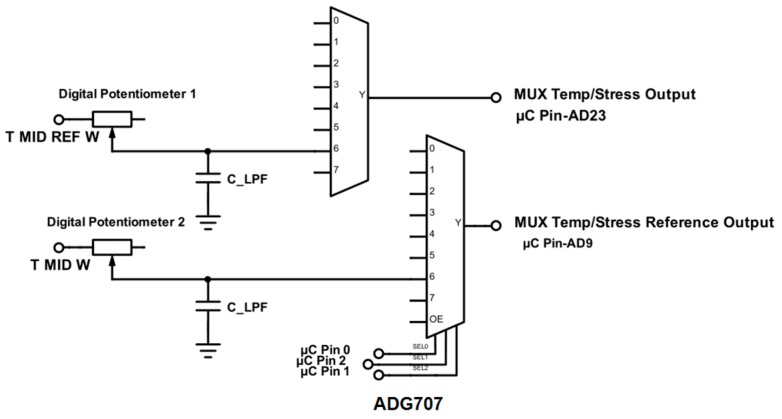
Circuit schematic showing a digitally controlled low-pass filter at each input stage for both sensor multiplexers.

**Figure 12:**
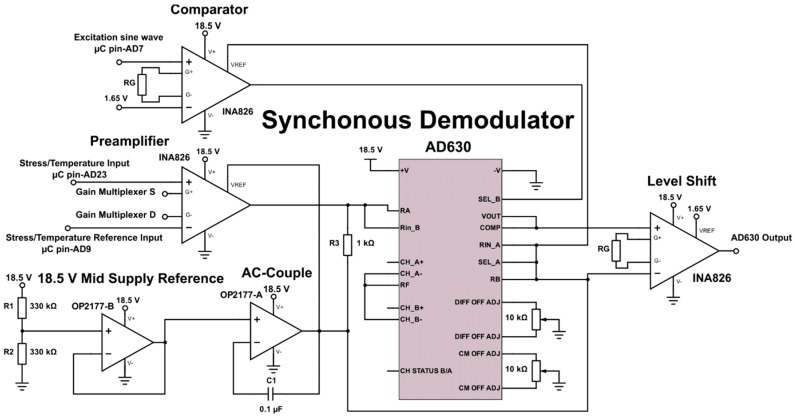
Circuit schematic of the AC coupled AD630 synchronous demodulator in a lock-in amplifier configuration.

To further increase the resolution of the calibration a low-pass filter was added to each output of the Wheatstone bridge (Figure 11). The AD630 IC (Analog Devices) is an established precision and reasonably low power (<5 mA) synchronous demodulator that can be set up in a lock-in amplifier configuration. In this configuration the device is able to recover signals from 100 dB of noise [27]. The AD630 was used in lock-in amplifier configuration (Figure 12). An INA826 (Texas Instruments) instrumentation amplifier (Figure 12) was used to multiply the sensor signals of the multiplexed Wheatstone bridge outputs of the temperature and stress sensors.

**Figure 13:**
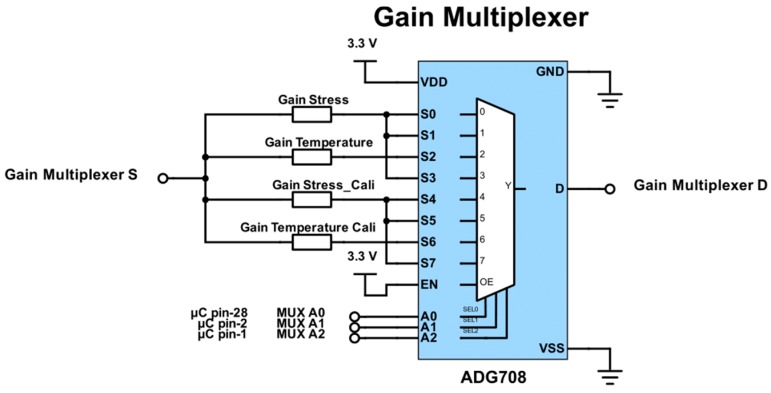
Circuit schematic of the preamplifier gain multiplexer.

AC coupling was provided for the preamplifier by using the feedback network of *R3*, *C1* and an OP2177 (Analog Devices) operational amplifier (Figure 12), which was biased at the high level mid-supply voltage (9.25 V) using another OP2177. This coupling reduced input referred noise with frequencies below the high-pass cut-off frequency (~1.6 kHz), therefore allowing small signals with higher frequencies to be measured that would otherwise be hidden in noise. This coupling also has the bonus of removing any offset error introduced by the preamplifier, thereby stabilizing the AD630 output. A second INA826 was used to level shift the comparator input of the excitation sine wave to the AD630. The reason behind choosing an identical amplifier to that of the preamplifier was to compensate for any phase difference that was introduced during the amplification stage. A third INA826 level shifted the output signal of the AD630 back to the 1.65 V bias level. The inclusion of two manual potentiometers allowed for the differential and common mode voltage offsets of the AD630 to be nulled. By multiplexing the gain of the preamplifier, different gains for the stress and temperature sensing can be selected (Figure 13). Importantly, a significantly lower calibration gain can be selected to calibrate the Wheatstone bridge arrangements.

**Figure 14:**
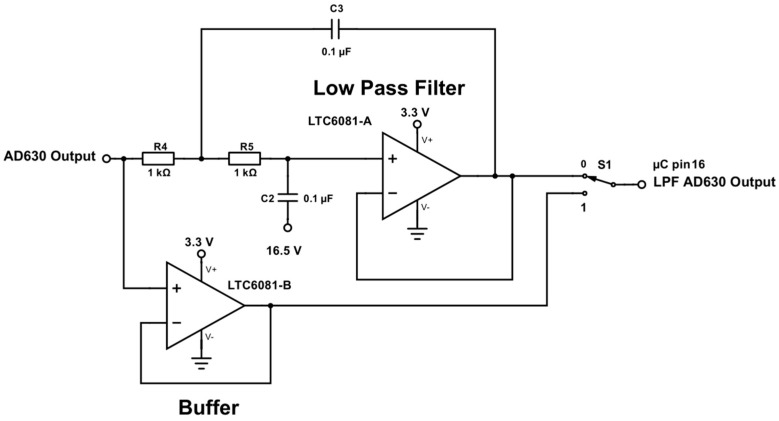
Circuit schematic showing the ADC buffering and low-pass filter stage of the AD630 output.

In the final stage the AD630 output was low-pass filtered with a unity gain second-order Sallen-Key filter and then buffered using an LTC6081 operational amplifier. Alternatively, the output can also be directly buffered with a different LTC6081, bypassing the low-pass filter, before being fed to the ADC (Figure 14).

### 2.4. Cyclic Voltammetry

**Figure 15:**
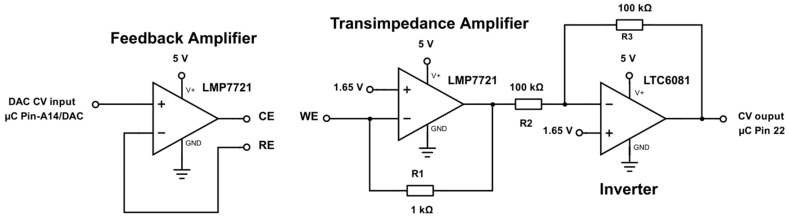
Circuit schematic showing a basic potentiostat setup for cyclic voltammetry interfacing to the WE, CE and RE.

A basic potentiostat design of the cyclic voltammetry circuit was chosen (Figure 15). The design has been adapted from literature [28]. As a very low input bias current is required, the selection of the feedback and transimpedance amplifier is of particular importance. The low input bias current is required to not polarize the reference electrode and to minimally interfere with the transimpedance current path. The selected LMP7721 (Texas Instruments) has excellent offset voltage characteristics and transient behaviour.

### 2.5. Voltage Transient Measurements

**Figure 16:**
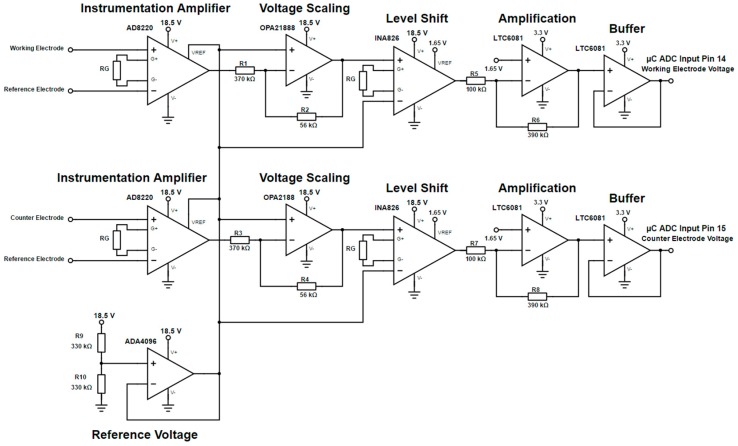
Circuit schematic for measuring the WE-RE and CE-RE transient voltages.

Another circuit has been designed (Figure 16) to provide a measurement of the transient voltage over the WE-RE and CE-RE pairs, respectively. The circuit chain consists of an AD8220 instrumentation amplifier to record the differential voltage, an OPA2188 to apply voltage scaling to reduce the voltage level to within ±1.5 V and an INA826 to level shift the signal to a 1.65 V bias. The final components in the signal chain are the LTC6081 to provide optional amplification and a signal buffer to prevent exceeding the input voltage limitation of the microcontroller’s ADC.

### 2.6. Power Consumption

The power consumption of the various sensor modules during operation was estimated. As the different sensor modules are not indented for continuous monitoring, but rather for short intermittent measurements over time, power consumption is less critical.

## 3. Results

### 3.1. pH Measurements

**Figure 17:**
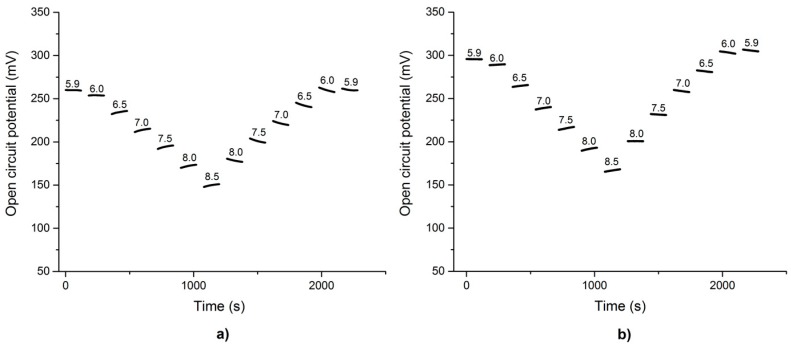
pH series from pH 5.9 to pH 8.5 in steps of 0.5 for an iridium oxide electrode vs. an Ag/AgCl reference electrode. For each pH measurement one minute was taken to swap the pH electrode and give the sensor time to adapt to the new environment, followed by two minutes to measure the sensors response. Measurement using (**a**) a digital multimeter (Agilent model 34405A) and (**b**) the custom-made pH readout system.

Open circuit potentials were measured with the pH sensors for a range of 5.9 to 8.5 in steps of 0.5 with a commercial digital multimeter and the custom pH readout systems (Figure 17a,b). The measurements show no significant difference in the average open circuit potential between the two recordings, with the recording of the custom front-end appearing to be more stable.

**Figure 18:**
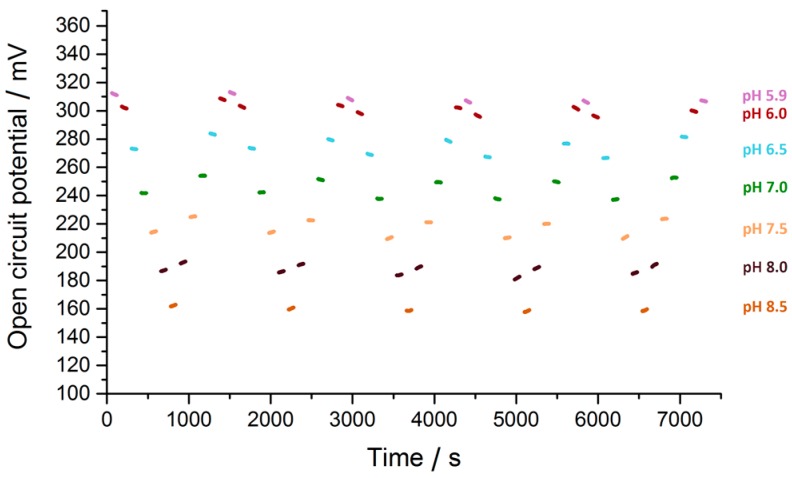
pH value series from pH 5.9 to pH 8.5 in steps of 0.5 for an iridium oxide electrode vs. an Ag/AgCl reference electrode. One minute of open circuit potential recording followed by one minute to change the pH solution and allow the sensor some time to adapt to the new solution. Data recorded using the custom made electronics.

Evaluation of stability and continuity of the sensors was performed applying the same pH level series (Figure 18). Sensor voltages followed the pH value and were reproducible over several cycles whereas hysteresis could be observed depending whether the pH value was increased or decreased, respectively.

**Figure 19:**
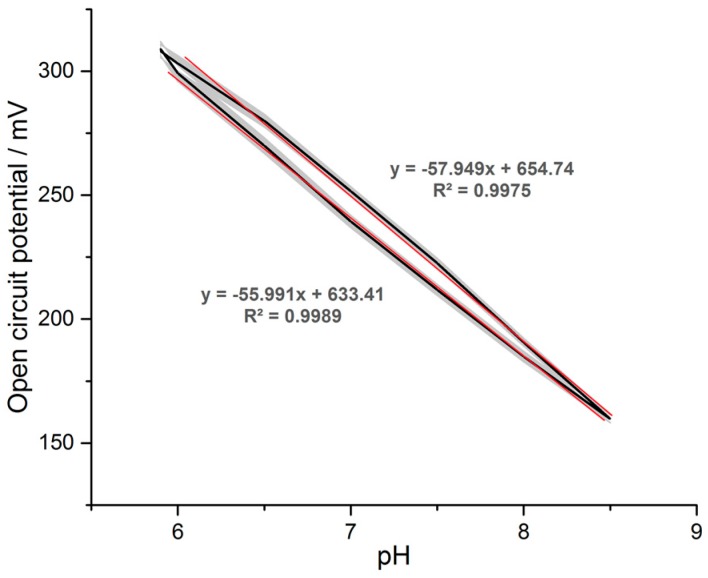
Hysteresis plot of the pH vs. open circuit potential recorded during the stability test. Grey area indicates the standard deviation of the repeated pH series with trend lines. The average Nernstian slope of the pH curve is −57.301 mV/pH.

The hysteresis curve (Figure 19) was extracted from these data and Nernstian slopes were calculated. Averaged the measurements result in a Nernstian curve of −57.301 mV/pH, which was within the range expected for a pH recording. Overall the repeatability error of the long term experiment was less than 1.4% and the resulting resolution less than 0.0002 pH units.

### 3.2. Impedance Spectroscopy

**Figure 20:**
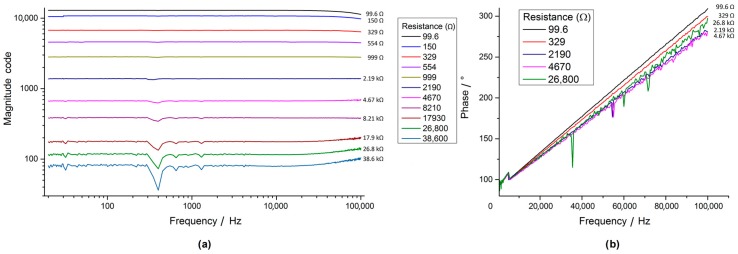
Characteristic curves of the AD5933 impedance converter setup for a selection of resistor values for (**a**) the magnitude code and (**b**) the measured system phase versus frequency. The graph in (**a**) shows significant resonance behaviour at 300 Hz, however the effect becomes less severe at lower resistance values.

Impedance spectroscopy delivered constant magnitude values over the frequency range (20 Hz to 100 kHz) with test resistors between 99.6 Ω and 38.6 kΩ at increasing amplitudes (Figure 20a) and a linearly increasing phase over the frequency showing slight dispersion (Figure 20b). A resonant behaviour at around 300–500 Hz with resistors below 1 kΩ was observed (Figure 20) in the magnitude spectrum.

**Figure 21:**
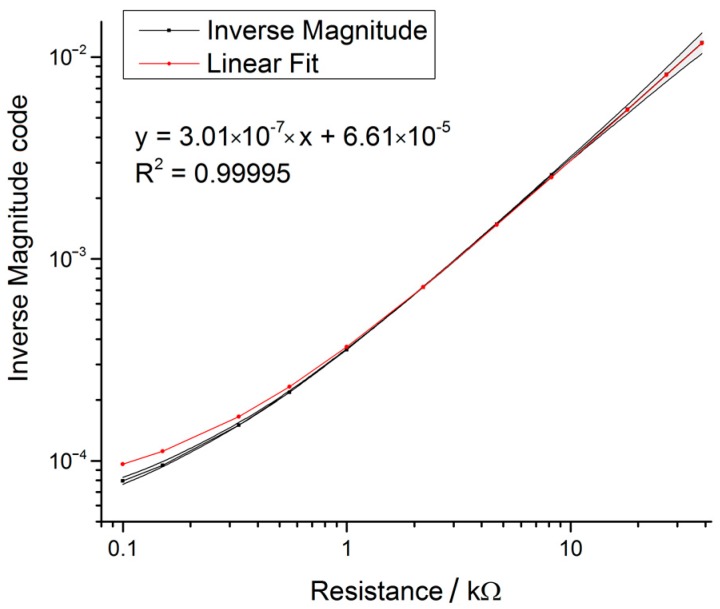
Inverse of the magnitude curve versus resistance showing the linear behaviour of the AD5933. In the range of 1 kΩ to 10 kΩ the system showed best linear behaviour and represented the optimal measurement range of the device setup.

The phase behaviour across the resistors was dependent on frequency, however, not on the actual resistance values (Figure 20). Furthermore, the phase behaviour was erratic and provided one of the largest sources of erroneous measurements and underlined the need to calibrate the phase for each measurement point. The inverse magnitude versus resistance characteristic over the selected E12 resistor range for calibration of the impedance measurement system was nearly linear on a double logarithmic scale (Figure 21).

By comparing the measured values of a series of E12 resistors using the AD5933 setup and the LCR meter, an average trueness of 99.78% was determined for the range of 1 kΩ to 10 kΩ (Table 4). Trueness has been chosen according to ISO 5725-1 as the closeness of the mean of a set of measurement data to the actual measured value. These values corresponded to the expected range of electrode resistance under investigation.

**Figure 22:**
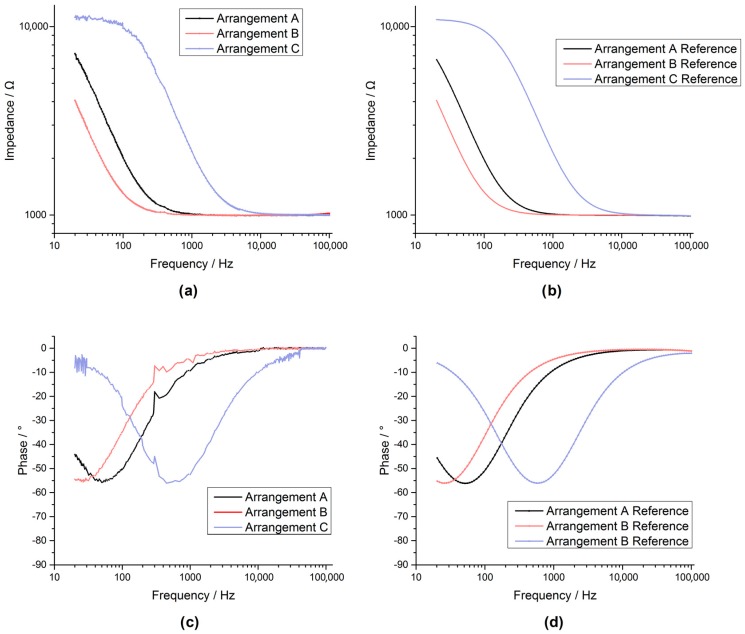
The Bode plot for the arrangements A, B, C recorded with the AD5933 custom system are shown in figures (**a**,**c**), while the bode plots for the same arrangements recorded by an Autolab PGSTAT302N impedance module are shown in figures (**b**,**d**).

**Figure 23:**
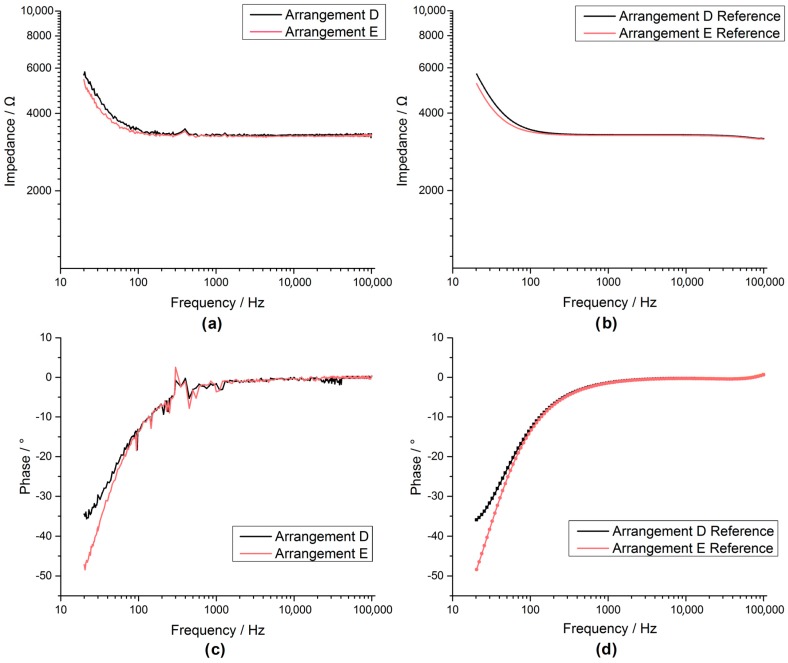
The Bode plot for the arrangements D, E recorded with the AD5933 custom system are shown in figures (**a**,**c**), while the bode plots for the same arrangements recorded by an Autolab PGSTAT302N impedance module are shown in figures (**b**,**d**).

The spectroscopy measurements (50 mV excitation at 20 Hz to 100 kHz) of the custom AD5933 system (Figure 22a,c) were comparable to the commercial reference system (Figure 23b,d) for arrangements A, B and C. This also held true for arrangements D and E (Figure 23) but showing artefact behaviour between 300 and 500 Hz.

By comparing the results (Figure 22 and Figure 23) between the system and reference measurements, it was evident that although the custom AD5933 system had considerable noise and distortion, it showed reasonable performance in tracking the impedance shape. The overall shape of the AD5933 recording could potentially be further improved by using a smoothing filter.

**Figure 24:**
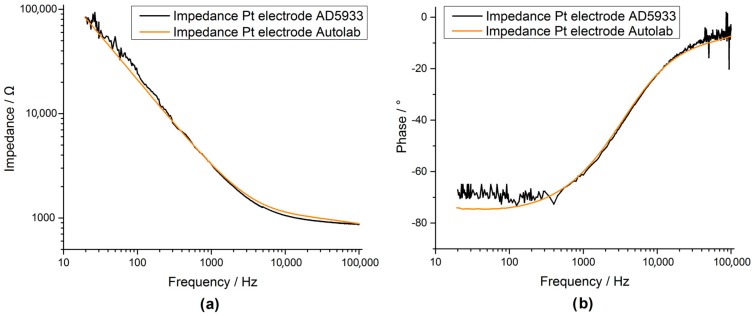
Bode plot of a small platinum electrode with a diameter of 0.85 mm (magnitude (**a**) and phase (**b**)). Measurement configured in a three electrode setup. For comparison both the measurement for the custom AD5933 system (black) and the Autolab system (orange) are shown.

Impedance spectroscopy of a real platinum electrode, using a three electrode setup (Figure 24), proved the performance of the custom system but exceeded the measurable range of the custom AD5933 system at low frequencies due to the high impedance of the electrode. To account for higher impedances, the transimpedance resistor (10 kΩ resistor in Figure 5) should be changed to a higher value to increase the gain of the amplifier system.

### 3.3. Temperature / Stress

**Figure 25:**
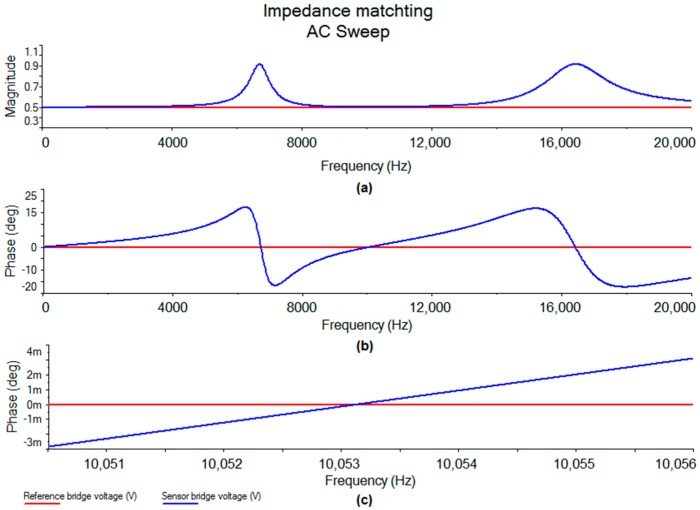
Simulation of an AC sweep across a balanced modified Wheatstone bridge showing (**a**) magnitude; (**b**) phase and (**c**) magnified phase at 10 kHz vs. applied AC frequency.

The simulation of the reactive correction of the Wheatstone bridge (Figure 25) showed compensation with a positive or negative phase correction, respectively. This correction enabled high precision due to the excitation frequency having a resolution of 1 Hz.

### 3.4. Power Consumption

The power consumption of the different sensor modules was in the range between 41 mW and 119 mW (Table 5).

## 4. Discussion and Conclusions

Although sophisticated electronics for electrochemical measurements are available, few options exist that allow combined measurement modalities simultaneously. Therefore, we developed a framework of such a system that is low cost (compared to commercially available electrochemical devices) and portable, allowing practical use of the system for animal studies.

Measuring changes in pH value caused by electrical stimulation is a complicated task. Three different factors could contribute to a falsified measurement. These include artefacts (generated from the stimulation pulse), sensor drift and the proximity of the pH sensing electrode to the stimulation electrode under investigation. By using a differential pH measurement of a pH sensitive electrode and a pH insensitive electrode (both relative to a common reference electrode), it is proposed that artefacts and sensor drift can be reduced. Furthermore, placing the pH electrodes closer to the stimulation electrode one can increase the accuracy of the measurement.

The pH readout system achieved an excellent resolution of <2 × 10^−4^ pH units and showed a repeatability error of less than 1.4% over a two-hour period in which the solution was alternated 61 times. Although hysteresis was present on the pH testing electrode, an average response of 57.301 mV/pH could be determined for a pH testing range of 5.9 to 8.5. Compared to a commercial digital multimeter (Agilent 34405A) the designed system showed comparable results and no deficit between the two systems was detected. This variation in the two different pH series (Figure 17) was most likely due to changes in the sensor. The hysteresis behaviour in each of the experiments was most likely due to different chemical reactions occurring on the electrode surface. Unlike conventional pH sensors implemented for in vivo applications, the proposed design made use of an amplifier with a high input voltage range capable of withstanding stimulation induced voltages.

With the electrode and electronic design presented in this paper, the feasibility of measuring pH within a stimulation pulse could be studied. Similarly, the challenge of finding the most appropriate pH reference electrode for these measurements can be investigated.

For impedance measurements, the use of different electrode configurations and arrangements can provide valuable insight into electrode characteristics and the surrounding biological environment. The proposed design suggests a method to obtain a two, three or four electrode impedance measurement as well as a small impedance mapping around the electrode. Eventually, this could provide enough insight to resolve the immune response to an implanted active device and the variability in scar tissue formation.

The AD5933 impedance sensor showed promising results compared to a commercial impedance spectrometer Figure 22, Figure 23 and Figure 24, however, to achieve a large measurable impedance span (allowing both large and small electrodes to be fully measured) the parameters of the IC (i.e., system gain, the PGA gain and voltage excitation levels) need to be carefully selected. The approach of using current excitation at high impedance values should help to expand the range considerably. The cause of the resonant behaviour experienced at around 300 to 400 Hz should be further investigated, but is most likely due to the PCB routing or originates from one opponent in the circuit chain.

The core functionality of the lock-in amplifier is shown in the datasheet [27]. The presented work outlines how this fundamental design can be adapted to collect measurement information from a variety of temperature and stress sensors in vivo, addressing the challenges created by stimulation induced artefacts and sensor drift. In particular, a solution was presented to calibrate an imbalanced Wheatstone bridge by changing the excitation frequency rather than using a potentiometer and therefore, allowing more accurate and faster calibration.

All electronic components used for the design are primarily chosen for their power efficiency, however, not at the expense of precision or key performances. Providing power to the system would therefore require the use of an implantable high capacity battery or inductive power transmission. Power consumption is nevertheless not expected to be critical for enabling the electrode measurements envisioned since the measurements are not intended to run continuously, but rather intermittently over a long period of time. This allows the focus to be placed on accuracy and precision rather than power consumption.

A modular approach to the design allows for a tailored system to be implemented for a specific task, therefore allowing for a decrease in size, power consumption and cost, depending on the sensors intended to be implemented. The sensor-array must be further developed and miniaturized before it can be used for in vivo applications. Combining the individual sensors and electrodes into a single electrode array can be achieved with micromachining technology [29,30].

The next steps in the development process will be to fully evaluate the framework with the sensor array and finalize the system for long term measurements and improving miniaturization of the final system. Furthermore, the cyclic voltammetry and transient measurement module still have to be developed as a prototype. The sensor system shall in future be integrated with an electrical stimulator, allowing the intended key experiments to be performed under stimulation conditions. The hope is that more detailed and comprehensive research will lead to the creation of proper design rules, paving the way for a new generation of long term stable neural electrode arrays.

## Figures and Tables

**Table 1:** External clock frequencies required to achieve a specific frequency range [25].

Clock Frequency	Frequency Range
16 MHz	5–100 kHz
4 MHz	1–5 kHz
2 MHz	300 Hz–1 kHz
1 MHz	200–300 Hz
250 kHz	100–200 Hz
100 kHz	30–100 Hz
50 kHz	20–30 Hz

**Table 2:** Excitation voltage amplitudes and DC bias levels for a supply voltage of 3.3 V [24].

Voltage Mode	Output Excitation Voltage Amplitude	Output DC Bias Level
1	1.98 V p-p	1.48 V
2	0.97 V p-p	0.76 V
3	383 mV p-p	0.31 V
4	198 mV p-p	0.173 V

**Table 3:** Truth table of the switching protocol for switches (1) to (5) of Figure 5. Electrode configurations allow for two, three and four electrode impedance measurements using current or voltage excitation, respectively.

Source	Voltage		Current	
Electrode Configuration	2	3	2	4
Switch (1)	0	0	0	1
Switch (2)	0	0	1	1
Switch (3)	0	1	0	0
Switch (4)	0	0	1	1
Switch (5)	0	1	1	1

**Table 4:** Precision in relative standard deviations (RSD) and trueness error (averaged accuracy error) of the impedance sensor over an entire frequency sweep to determine the resistance of a series of test resistances. A two-point calibration method was used and the resistances were measured using a 4-point LCR meter (Escort ELC-3131D).

Parameter	Resistance/Ω							
	995.1	1483	2698	3892	5602	6769	8251	9772
Precision (RSD %)	0.144	0.283	0.865	1.29	1.826	1.638	2.607	2.810
Trueness error (%)	−0.456	−0.03	−0.274	0.172	−0.250	0.299	−0.121	−0.138

**Table 5:** Estimated power consumption of the various sensor modules.

Module	Power Consumption/mW
pH (Single)	58.0
Impedance	48.9
Stress/Temperature	119.0
Cyclic Voltammetry	41.1
Transient Measurements	72.4

## Abbreviations

The following abbreviations are used in this manuscript.

ADC	Analog-to-Digital Converter
WE	Working Electrode
CE	Counter Electrode
RE	Reference Electrode
CV	Cyclic Voltammetry
Ag	Silver
Cl	Chloride
Pt	Platinum
Ir	Iridium
